# The Epidemic of Sexually Transmitted Diseases Under the Influence of COVID-19 in China

**DOI:** 10.3389/fpubh.2021.737817

**Published:** 2021-12-16

**Authors:** Xiangyu Yan, Xuechun Wang, Xiangyu Zhang, Lei Wang, Bo Zhang, Zhongwei Jia

**Affiliations:** ^1^School of Public Health, Peking University, Beijing, China; ^2^Taiyuan Center for Disease Control and Prevention, Taiyuan, China; ^3^Center for Intelligent Public Health, Institute for Artificial Intelligence, Peking University, Beijing, China; ^4^Center for Drug Abuse Control and Prevention, National Institute of Health Data Science, Peking University, Beijing, China

**Keywords:** epidemic, sexually transmitted diseases, HIV/AIDS, hepatitis, gonorrhea, syphilis, COVID-19

## Abstract

**Background:** Prevention and control of HIV/AIDS and other sexually transmitted diseases (STDs) are major public health priorities in China, but are influenced by the COVID-19 epidemic. In this study, we aimed to quantitatively explore the impact of the COVID-19 epidemic and its control measures on five major STD epidemics in China.

**Methods:** A monthly number of newly reported cases of HIV/AIDS, hepatitis B and C, gonorrhea, and syphilis from January 2010 to December 2020 were extracted to establish autoregressive integrated moving average (ARIMA) models. Each month's absolute percentage error (APE) between the actual value and model-predicted value of each STD in 2020 was calculated to evaluate the influence of the COVID-19 epidemic on the STDs. Pearson correlation analysis was conducted to explore the confirmed COVID-19 case numbers and the COVID-19 control measures' correlations with the case numbers and the APEs of five STDs in 2020.

**Results:** The actual number of five STDs in China was more than 50% lower than the predicted number in the early days of the COVID-19 epidemic, especially in February. Among them, the actual number of cases of hepatitis C, gonorrhea, and syphilis in February 2020 was more than 100% lower than the predicted number (APE was −102.3, −109.0, and −100.4%, respectively). After the sharply declines of STDs' reported cases in early 2020, the case numbers recovered quickly after March. The epidemic of STDs was negatively associated with the COVID-19 epidemic and its control measures, especially for restrictions on gathering size, close public transport, and stay-at-home requirements (*p* < 0.05).

**Conclusion:** COVID-19 had a significant but temporary influence on the STD epidemic in China. The effective control of COVID-19 is vital for STD prevention. STD services need to be improved to prevent STDs from becoming a secluded corner in the shadow of COVID-19.

## Introduction

The pandemic of COVID-19 has brought great challenges to the world, and up to now, the number of COVID-19 cases has exceeded 130 million worldwide, involving more than 200 countries and regions (1). In response to the severe situation of COVID-19, countries have taken various prevention and control measures, such as temporarily closing borders, suspending school work, social isolation measures, and redistributing medical resources. In the early stage, China adopted strict and effective restrictive measures to control the spread of COVID-19, which began from the lockdown of Wuhan on January 23, 2020, to the resumption of work and production in early March, and then normal prevention and control measures for COVID-19 was implemented around China (2).

The previous studies had shown that the containment and closure policies were of great value for effective control of COVID-19's spread (3). However, these measures that were aimed at COVID-19 prevention and control also had impacts on the daily prevention and control work of other infectious diseases, especially for sexually transmitted diseases (STDs). The control of STDs is always a difficult task because it is not only a medical issue, but also a social issue for the related stigma and discrimination. During the epidemic of COVID-19, the control of STDs became a secluded corner overlooked. Recent studies indicated that the epidemic of COVID-19 and the control policies during the lockdown period could impact the epidemic of STDs (4–8) and related medical services around the world (9–13). However, the COVID-19 epidemic and its control measures' influence on the epidemic of STDs in China has not been quantitatively evaluated yet. There are five major STDs among the 40 notifiable infectious diseases that caused a high burden on the public health, such as HIV/AIDS, hepatitis B, hepatitis C, gonorrhea, and syphilis (14). In this study, we aimed to explore the epidemic of the five major STDs under the influence of COVID-19 in China in 2020, and the containment and closure policies' effects on the STD epidemic.

## Methods

### Data Source

The monthly number of newly reported cases of HIV/AIDS, hepatitis B, hepatitis C, gonorrhea, and syphilis from January 2010 to December 2020 were extracted from the website of the Bureau for Disease Control and Prevention of China National Health Commission (http://www.nhc.gov.cn/jkj/new_index.shtml). The data published by the government were extracted from the routine reporting system for notifiable infectious diseases covered 31 provinces in the Chinese mainland, which was established by the Chinese government in the 1950s and switched from paper-based reporting to web-based reporting since 2003 (15). The case information of notifiable infectious diseases was timely reported from local hospitals and community health service centers throughout the country and was reviewed and confirmed by local Centers for Disease Control and Prevention (CDC) after confirmatory tests (15). Nowadays, a total of 40 kinds of notifiable infectious diseases are monitored by this system, among which there are five kinds of STDs, such as HIV/AIDS, hepatitis B, hepatitis C, gonorrhea, and syphilis (14). The Chinese government publishes the statistical table for reported case numbers of notifiable infectious diseases nationwide monthly on the government's website without detailed sociodemographic characteristics of cases.

The daily confirmed COVID-19 case numbers of China in 2020 were extracted from the COVID-19 data repository provided by Johns Hopkins University (16). The indicators of government control measures were extracted from the Oxford COVID-19 Government Response Tracker (OxCGRT) (17). Among the OxCGRT indicator system, seven indicators that reflected government response on “lockdown” restrictions, containment, and closure policies were extracted daily at the provincial level in China in 2020, such as school closing, workplace closing, cancel public events, restrictions on gathering size, close public transport, stay at home requirements, and restrictions on internal movement (17). The higher score of these indicators, the related policies were more strictly implemented. In addition, the dates of each province's start and end of lockdown period were obtained from each province's official press conferences or websites. The lockdown period of a province was considered as the period of provincial government's Level I response to a major public health emergency, which was the highest level of infectious disease control response, such as containment and closure policies (18).

This study was the secondary analysis of online reports data and other publicly available data and did not access any individually identifiable data. Therefore, ethical review was waived.

### Statistical Analysis

#### Model Construction and Prediction

Considering the long-term rising trend and periodicity of the STD epidemic (i.e., HIV/AIDS) in China (19), except comparing the STD case numbers in 2020 with that of the same period in previous years, autoregressive integrated moving average (ARIMA) models were also established based on previous STD data, and to “forecast” the number of newly reported STDs cases in 2020. Then, the influence of COVID-19's epidemic on the five STDs could be better evaluated by comparing each month's actual number of STD cases in 2020 with the predicted number forecasted by the models.

Given the 12-month seasonality of the number of newly reported cases of STD (19), seasonal ARIMA models were used to formulate the long-term trend of these STDs. The *forecast::auto.arima()* function in R environment was used to choose the optimal set of parameters, which could compare the combinatorial spectrum of parameters based on the rule of the minimum Akaike's information criterion (AIC) and Bayesian information criterion (BIC). Ljung–Box test was used to check whether the residual sequence was a white noise sequence (*p* > 0.05 for the white noise sequence). Diagnostic checking parameters such as root mean square error, mean absolute percentage error (MAPE), AIC, and BIC were used to evaluate the goodness-of-fit of constructed models. We used the number of newly reported STD cases from January 2010 to December 2019 to construct the ARIMA models, and then forecasted the STD case numbers of January 2020 to December 2020. Each month's absolute percentage error (APE) in 2020 of each disease was calculated to evaluate the influence of COVID-19's epidemic, which was calculated as the following formula:


$$\begin{array}{l} APE=\frac{\left(Number_{actual\;reported\;cases}-Number_{predicted\;cases\;by\;ARIMA}\;\right)}{\;Number_{actual\;reported\;cases}} \end{array}$$

A sensitivity analysis that used the data from January 2010 to December 2018 for model construction, and forecasted the case numbers of January 2019 to December 2020 was conducted to verify the results.

#### Correlation Analysis

The average scores among the 31 provinces in each day of the seven OxCGRT indicators were calculated, respectively, to form daily national indicators of COVID-19 control measures. And monthly average scores of the seven national control measures indicators and monthly cumulative number of confirmed COVID-19 cases were also calculated for the correlation analysis. Pearson correlation analysis was conducted to explore monthly confirmed COVID-19 case numbers and the COVID-19 control measures' correlations with the case numbers and the APEs of five STDs in 2020. The Pearson correlation coefficient (r) was calculated to reflect the correlations. Considering the specificity of February, when the number of COVID-19 cases was highest and control measures were strongest, we additionally conducted correlation analysis using the 11 months' data except February to explore correlations in the situation of normal COVID-19 control measures.

A two-sided *p* value of 0.05 was used for null-hypothesis tests. Statistical analyses were conducted using R version 4.0.3 (R Core Team).

## Results

### Epidemic of Five STDs

**Figure 1:**
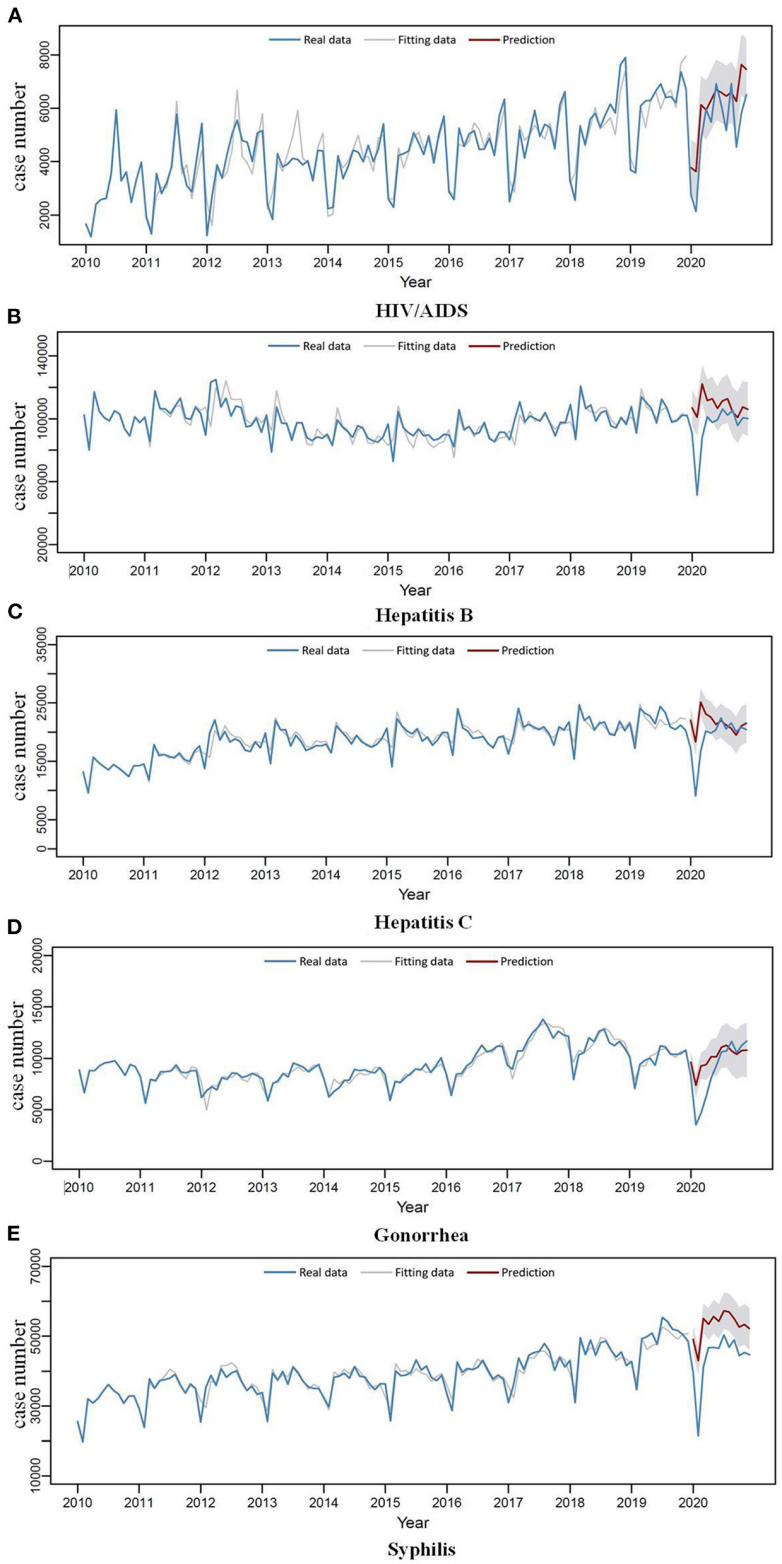
Autoregressive integrated moving average model fitting and prediction of the newly reported case number of five STDs. **(A)** HIV/AIDS. **(B)** Hepatitis B. **(C)** Hepatitis C. **(D)** Gonorrhea. **(E)** Syphilis.

The case numbers of HIV/AIDS and other four STDs showed sharp declines in early 2020, especially in February, compared to the same period in previous years. And the reported cases of hepatitis B, hepatitis C, and gonorrhea in February 2020 were the lowest among the 11 years (Figure 1; Supplementary Figure 1).

The optimal ARIMA models of each STD were listed in Table 1, and the optimal models of the sensitivity analysis were listed in Supplementary Table 1. The MAPE of each model was <10% and had passed the Ljung–Box test, which showed a good fitting effect (Table 1; Supplementary Table 1). *Via* comparing the actual and predicted number of cases of five STDs in 2020, it showed that the actual case numbers of the five STDs in the early period of the COVID-19 epidemic in China were more than 50% lower than the predicted number, especially in February 2020 (Figure 1; Table 2; Supplementary Figure 1; Supplementary Table 2). Among them, the actual case numbers of hepatitis C, gonorrhea, and syphilis in February 2020 were more than 100% lower than the predicted number, and the APE was −102.3, −109.0, and −100.4% for the three STDs, respectively (Table 2). The sensitivity analysis showed similar results (Supplementary Table 2).

**Table 1:** Parameters and goodness-of-fit of the five STDs' optimal ARIMA models.

**Disease**	**Optimal model**	**Goodness-of-fit**				**Ljung-Box test**	
		**RMSE**	**MAPE (%)**	**AIC**	**BIC**	***χ^2^* value**	***P* value**
AIDS	ARIMA(1,0,1) × ${\left(0,1,1\right)}_{12}^{*}$	489.07	9.04	1668.14	1681.55	0.010	0.92
Hepatitis B	ARIMA(3,1,0) × (2,1,0)_12_	5447.44	3.62	2179.39	2195.43	0.000	0.99
Hepatitis C	ARIMA(3,1,0) × (2,1,2)_12_	1031.85	3.82	1842.22	1863.6	0.005	0.94
Gonorrhea	ARIMA(0,1,3) × (0,1,1)_12_	507.16	3.91	1673.12	1686.48	<0.001	0.98
Syphilis	ARIMA(2,1,2) × (0,1,1)_12_	1794.99	3.39	1950.41	1966.45	0.002	0.96

*RMSE, root mean square error; MAPE, mean absolute percentage error; AIC, Akaike's information criterion; BIC, Bayesian information criterion*.

* Draft was included in the model.

**Table 2:** Each month's actual case number, predicted number, and absolute percentage error (APE) of five STDs in 2020.

**Month**	**HIV/AIDS**			**Hepatitis B**			**Hepatitis C**			**Gonorrhea**			**Syphilis**		
	**Actual**	**Predicted**	**APE (%)**	**Actual**	**Predicted**	**APE (%)**	**Actual**	**Predicted**	**APE (%)**	**Actual**	**Predicted**	**APE (%)**	**Actual**	**Predicted**	**APE (%)**
January	2759	3781.3	−37.1	91026	106952.2	−17.5	17287	22031.6	−27.4	8254	9619.2	−16.5	39671	49093.1	−23.8
February	2133	3627.5	−70.1	51506	100954.5	−96.0	9068	18342.0	−102.3	3524	7366.5	−109.0	21448	42986.4	−100.4
March	4808	6130.3	−27.5	88150	122105.0	−38.5	16718	25140.0	−50.4	4661	9263.9	−98.8	41154	55091.6	−33.9
April	5960	5932.3	0.5	101262	111610.1	−10.2	20179	23117.7	−14.6	6267	9383.0	−49.7	46728	53430.5	−14.3
May	5484	6309.9	−15.1	97651	112703.8	−15.4	19821	22561.0	−13.8	8104	10163.6	−25.4	46753	55654.1	−19.0
June	6915	6694.6	3.2	99319	106588.7	−7.3	20367	21291.8	−4.5	9292	10142.7	−9.2	46538	54259.7	−16.6
July	6124	6588.8	−7.6	106135	111396.0	−5.0	22400	21955.1	2.0	10621	11071.4	−4.2	50386	57257.6	−13.6
August	5166	6454.0	−24.9	102304	112745.1	−10.2	20520	21206.9	−3.3	10724	11271.8	−5.1	46838	56942.5	−21.6
September	6927	6603.4	4.7	105377	104300.9	1.0	21538	20641.9	4.2	11643	10716.5	8.0	48965	55047.8	−12.4
October	4546	6254.2	−37.6	95633	100812.5	−5.4	20067	19478.8	2.9	10551	10382.4	1.6	44438	52621.0	−18.4
November	5824	7635.7	−31.1	100561	107473.1	−6.9	20801	21022.6	−1.1	11260	10761.5	4.4	45305	53374.2	−17.8
December	6508	7451.9	−14.5	100209	106030.0	−5.8	20438	21513.8	−5.3	11691	10796.4	7.7	44696	52176.5	−16.7

After sharp declines of STD reported cases in early 2020, the case numbers recovered quickly, and then basically returned to normal. Among them, the reported cases numbers of HIV/AIDS experienced a trough from January to March 2020 and began to rise in April. The reported case numbers of hepatitis B, hepatitis C, and syphilis were at a low point only in February 2020, the reported cases began to rise in March and basically returned to the normal level in April. After reaching the bottom in February 2020, the reported case number of gonorrhea recovered relatively slowly, and basically returned to the normal level in June 2020 (Figure 1; Table 2; Supplementary Figure 1; Supplementary Table 2).

### Results of Correlation Analysis

**Figure 2:**
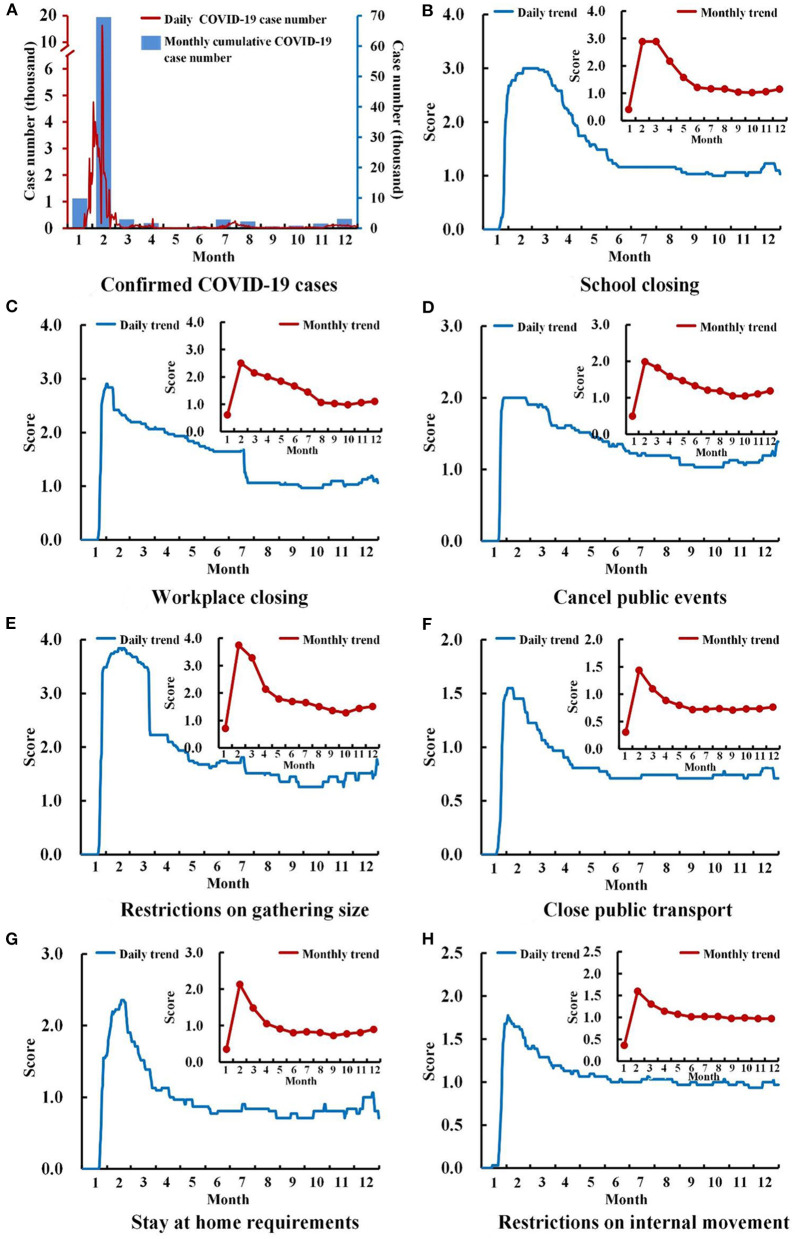
Trends of COVID-19 case numbers and scores of seven COVID-19 control measures in 2020. **(A)** Confirmed COVID-19 cases. **(B)** School closing. **(C)** Workplace closing. **(D)** Cancel public events. **(E)** Restrictions on gathering size. **(F)** Close public transport. **(G)** Stay at home requirements. **(H)** Restrictions on internal movement.

**Figure 3:**
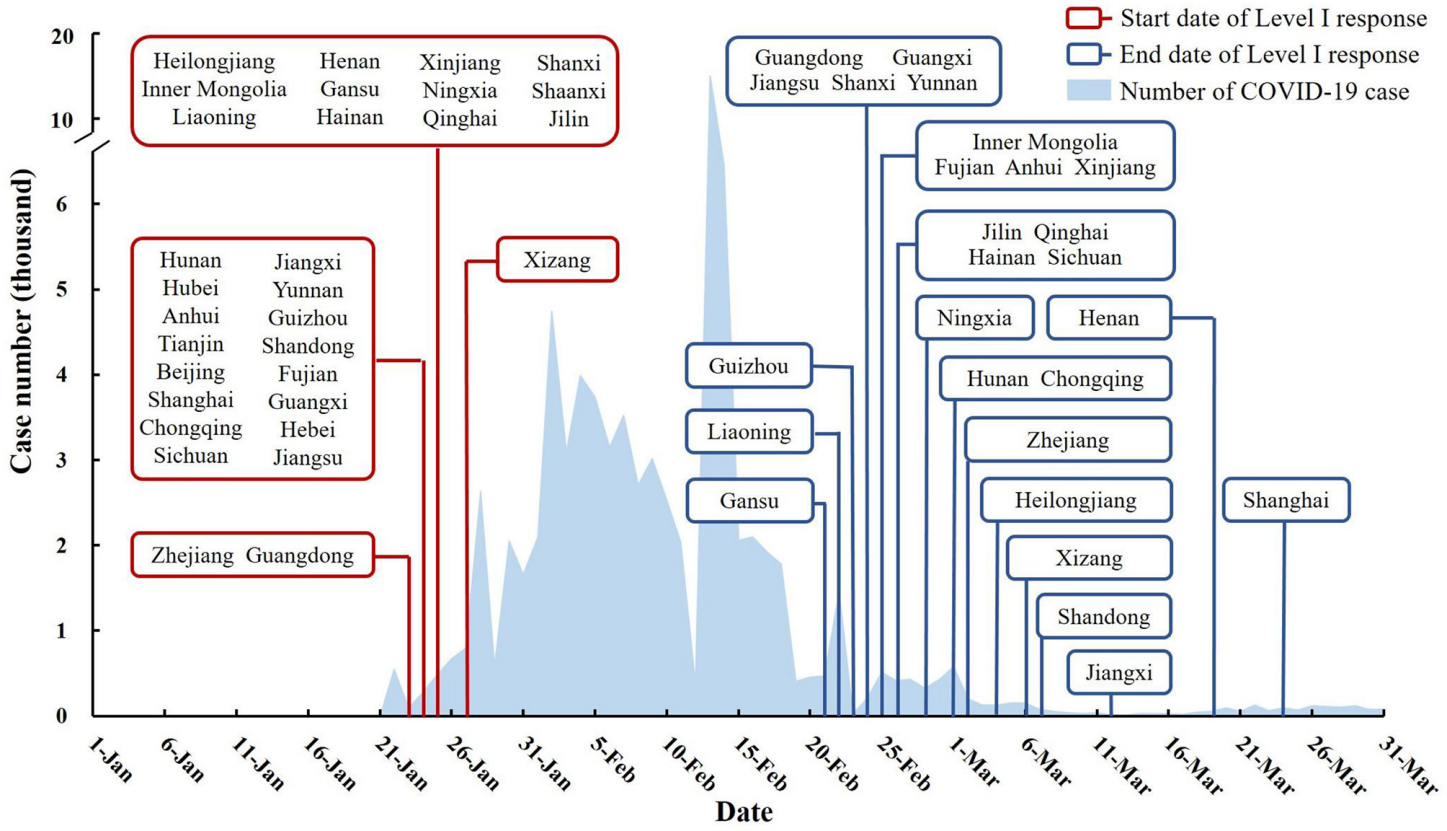
Lockdown period of different provinces in China.

The cumulative number confirmed COVID-19 cases dropped rapidly after peaking at 69,554 in February 2020 (Figure 2A). The lockdown periods of different provinces mainly concentrated in February, with the beginning of Level I response in late January and ending in late February to early March (Figure 3). The scores of seven national control measures indicators were also the highest in February (Figures 2B–H).

The monthly cumulative number of COVID-19 cases was negatively associated with the case numbers and APEs of the five STDs significantly (*p* < 0.05) (Table 3). All of the seven government control measures could influence the epidemic of STDs. Among them, the influence of restrictions on internal movement was relatively slight, which only negatively associated with APE of hepatitis B (*r* = −0.64, *p* < 0.05), gonorrhea (*r* = −0.67, *p* < 0.05), and syphilis (*r* = −0.62, *p* < 0.05) (Table 3). On the contrary, restrictions on gathering size, close public transport, and stay at home requirements had wider influences, of which the corrections with case numbers and APEs of hepatitis B, hepatitis C, gonorrhea, and syphilis were all significantly negative (*p* < 0.05) (Table 3). In addition, after excluding February, two indicators also influenced the APE of hepatitis B, hepatitis C, and gonorrhea, such as school closing (*r*_*hepatitisB*_ = −0.67, *p* < 0.05; *r*_*hepatitisC*_ = −0.62, *p* < 0.05; *r*_*gonorrhea*_ = −0.88, *p* < 0.001), and restrictions on gathering size (*r*_*hepatitisB*_ = −0.69, *p* < 0.05; *r*_*hepatitisC*_ = −0.62, *p* < 0.05; *r*_*gonorrhea*_ = −0.85, *p* < 0.001). Except for the above two indicators, the epidemic of gonorrhea was also influenced by workplace closing, canceling public events, and stay-at-home requirements considering its relatively slow recovery (*p* < 0.05) (Table 3).

**Table 3:** The correlation coefficients (r) of COVID-19 case numbers and control measures with the case numbers and the APEs of five STDs in 2020.

	**HIV/AIDS**				**Hepatitis B**				**Hepatitis C**				**Gonorrhea**				**Syphilis**			
**Variable**	**February**		**February**		**February**		**February**		**February**		**February**		**February**		**February**		**February**		**February**	
	**included**		**not included**		**included**		**not included**		**included**		**not included**		**included**		**not included**		**included**		**not included**	
	**Case number**	**APE**	**Case number**	**APE**	**Case number**	**APE**	**Case number**	**APE**	**Case number**	**APE**	**Case number**	**APE**	**Case number**	**APE**	**Case number**	**APE**	**Case number**	**APE**	**Case number**	**APE**
Number of COVID-19 cases	−0.72^**^	−0.75^**^	−0.74^**^	−0.46	−0.95^***^	−0.93^***^	−0.46	−0.32	−0.92^***^	−0.89^***^	−0.55	−0.45	−0.62^*^	−0.66^*^	−0.18	−0.10	−0.95^***^	−0.98^***^	−0.65^*^	−0.38
School closing	−0.28	−0.32	0.15	0.17	−0.63^*^	−0.74^**^	−0.31	−0.67^*^	−0.64^*^	−0.75^**^	−0.35	−0.62^*^	−0.84^***^	−0.92^***^	−0.76^**^	−0.88^***^	−0.56	−0.65^*^	−0.08	−0.46
Workplace closing	−0.17	−0.17	0.32	0.42	−0.58^*^	−0.69^*^	−0.14	−0.50	−0.57	−0.68^*^	−0.15	−0.45	−0.82^***^	−0.84^***^	−0.72^*^	−0.75^**^	−0.48	−0.60^*^	0.14	−0.22
Cancel public events	−0.10	−0.20	0.42	0.35	−0.54	−0.66^*^	−0.06	−0.45	−0.53	−0.63^*^	−0.07	−0.36	−0.78^**^	−0.79^**^	−0.56	−0.68^*^	−0.45	−0.59^*^	0.19	−0.24
Restrictions on gathering size	−0.35	−0.42	0.19	0.16	−0.73^**^	−0.83^***^	−0.31	−0.69^*^	−0.73^**^	−0.83^***^	−0.33	−0.62^*^	−0.82^***^	−0.92^***^	−0.70^*^	−0.85^***^	−0.67^*^	−0.77^**^	−0.07	−0.51
Close public transport	−0.27	−0.43	0.42	0.23	−0.71^**^	−0.79^**^	−0.04	−0.39	−0.68^*^	−0.74^**^	−0.02	−0.28	−0.67^*^	−0.79^**^	−0.42	−0.60	−0.64^*^	−0.77^**^	0.18	−0.26
Stay at home requirements	−0.42	−0.53	0.27	0.14	−0.81^***^	−0.89^***^	−0.23	−0.59	−0.79^*^	−0.85^***^	−0.23	−0.50	−0.76^**^	−0.86^***^	−0.57	−0.75^**^	−0.76^**^	−0.86^***^	−0.02	−0.44
Restrictions on internal movement	−0.07	−0.26	0.57	0.35	−0.54	−0.64^*^	0.16	−0.20	−0.49	−0.57	0.20	−0.07	−0.54	−0.67^*^	−0.26	−0.43	−0.54	−0.62^*^	0.39	−0.07

* *p < 0.05*.

** *p < 0.01*.

*** *p < 0.001*.

## Discussion

At the beginning of the COVID-19 epidemic and the lockdown in China, we found a short, and sharp decline in the reported case number of the HIV/AIDS and other four STDs, especially in February 2020. Due to the effects of the annual Chinese New Year, such as holiday for staff of hospitals and CDCs and decrease in people's willingness to seek medical services during the holiday, the decline of STD case numbers in January/February, represented by HIV/AIDS, was consistently observed (19). However, the actual case numbers in February 2020 were sharply lower than that of previous years and the predicted case numbers, which showed the significant influence of the COVID-19 epidemic and its control measures. The number of STD cases in other countries also decreased during the lockdown period, such as the gonorrhea cases in London, United Kingdom (4), syphilis cases in Rome, Italy (5) and the United States (6), gonorrhea and syphilis cases in Greece (7) and Madrid, Spain (8).

There might be three reasons for the decline of STD cases. Firstly, the containment and closure policies, as well as the health services' priority to COVID-19 controlled to the disruption of STDs testing and treatment. In this study, we found that the more stringent the control measures, the more declines in reported STD cases, especially for restrictions on gathering size, close public transport, and stay at home requirements, which brought obstacles for patients with STD to seek medical care because they had a lower priority compared to COVID-19 cases. A survey in China showed over one-third of people living with HIV faced the risk of antiretroviral treatment interruption, especially for patients who stayed in areas that implemented citywide lockdowns or travel restrictions, and rural areas (9). A similar phenomenon that the COVID-19 epidemic impacted routine STD services also occurred in other countries. In the United States, chlamydia and gonorrhea testing decreased 59% for female patients and 63% for male patients during the rapid spread of COVID-19 (10). A 60% reduction of STD screening in the COVID-19 evolving phase and a 100% reduction in the COVID-19 plateau phase were also observed in Rhode Island (13). In Melbourne, Australia, the STD consultations and services dropped more than 40% during lockdown (12). Whitlock et al. (4) and Crane et al. (6) showed the decrease of STD cases mainly because of a sharp drop in the number of asymptomatic cases in the United Kingdom, which were often diagnosed through screening programs, but the programs were particularly influenced by the healthcare disruptions during the lockdown. The perspective was supported by the study in Australia (12). The second reason was that the COVID-19 epidemic and lockdown affected the willingness of STD high-risk population's visits to health services, such as concerns about COVID-19 infection at the time of treatment (5), and the shame and fear of their families knowing about their infection status. The decline in STD clinic visits was common during the lockdown. Berzkalns et al.'s (11) study showed the sexual health clinic visits during lockdown were 55% lower than in 2019. More sharply, Tao et al.'s (13) study observed a 55% reduction in the number of STD clinic visits in the COVID-19 evolving phase and an 84% reduction in the COVID-19 plateau phase. The third reason was that the lockdown policies impacted the seeking of casual sexual partners and high-risk sexual behaviors. Though it was controversial by the United Kingdom and Denmark's studies (4, 20), a study conducted in Amsterdam showed a 73% reduction in the number of casual sex partners of men who have sex with men (21). Li et al.'s (22) survey also observed a decrease in the number of sexual partners and sexual frequency during the lockdown period in China, especially among people with high-risk sexual behaviors, which could effectively reduce the spread of STDs. The phenomenon was closely related to the containment and closure policies. Restrictions on gathering size led to the closure of bars and other entertainment venues, and closing public transport and stay-at-home requirements made it difficult for casual sexual partners to meet. In addition, a growing trend of STDs, represented by HIV, was observed among college students in China in recent years (23, 24). Continued school closing kept students at home and also could reduce the STD risk among college students.

Gradually, after March 2020, the epidemic of COVID-19 was under control to some extent in China, daily life, production, and medical services were carried out normally, and the number of STD cases rapidly returned to normal. This phenomenon indicated that the severe impact of COVID-19 on STDs in China was relatively short-lived, and the effective control of COVID-19 was the key to the recovery of STD prevention and control. It was related to the relatively relaxed containment and closure policies in the normal COVID-19 control period after February. A similar trend was also reported in the United States that after the “Stay Home, Stay Healthy (SHSH)” order, the weekly cases of gonorrhea and syphilis decreased, but returned to the pre-SHSH levels after reopening (11). The recovery might be partly due to increased testing as Chow et al.'s study (12) reported that asymptomatic screening gradually increased during post-lockdown.

However, in this study, we found how quickly the recovery of different STD case numbers varied. Unlike the other four diseases' rapid rebound, the recovery of gonorrhea's case number was relatively slow, which could be explained by these pathogens' different characteristics of the pathogenic process. For HIV, some atypical flu-like symptoms may occur in 2–4 weeks after infection, but infected people may not feel sick or ignore the symptoms (25). There is a relatively longer window period of HIV detection, especially for antibody tests with a window period of 23–90 days (26). Most chronic hepatitis B patients are asymptomatic, and the incubation period of hepatitis B is about 90 days (27). The newly acquired hepatitis C patients usually are asymptomatic or with mild symptoms that are likely to be ignored, and the incubation period is about 2–12 weeks (28). The signs and symptoms of primary and secondary syphilis are always mild and are unlikely to be noticed with a 3-month incubation period (29, 30). Therefore, the late diagnosis of these diseases was common. The rate of late diagnosis for HIV in developed countries was 24–30%, which was much higher in some developing countries (31). In China, the rate of late diagnosis for HIV was between 35.5 and 42.1% from 2010 to 2014 (32). Among hepatitis C patients, late diagnosis was common because 50–80% of them were unaware of their illness (33). In a study conducted in South China, delayed diagnosis occurred in about 78% of patients with syphilis (34). However, compared with the above four STDs, the incubation period of gonorrhea is much shorter for 1–14 days (35). Symptoms and signs of gonorrhea are more typical and easy to be noticed, such as dysuria, white, yellow, or green urethral discharge, and testicular or scrotal pain, which can promote patients to seek medical treatment as soon as possible and reduce late diagnosis (35). Therefore, due to the phenomenon of common late diagnosis and relatively longer incubation period, many of the HIV/AIDS, hepatitis B, hepatitis C, and syphilis patients detected in March were infected before the lockdown. There still was a large population of patients in incubation period or undetected status during the lockdown period. Hence, a rapid return to normal occurred after the lockdown period was observed. However, with a relatively lower proportion of patients in incubation period or undetected status, the relatively slow recovery of gonorrhea cases reflected that the number of newly infected patients during the lockdown really decreased, and gradually increased with the less strict policies implemented.

Though, on the whole, the number of STD cases in China declined in 2020, we must not let down our guard. UNAIDS has warned that the crisis of the COVID-19 pandemic had the potential to blow us even further toward the goal of ending AIDS by 2030, and the increased risk of COVID-19-associated adverse socioeconomic impacts could increase the high-risk populations' vulnerability to HIV (36, 37). Therefore, the long-term impact of the COVID-19 epidemic on STDs needs further attention and evaluation. And some non-contact STD testing and treatment services, such as mailing the testing kits and therapeutic drugs, need further development and improvement to prevent the STDs from becoming a secluded corner in the shadow of COVID-19.

This study had several limitations. First, because the data we collected from the government's report was the monthly total numbers of the STD cases, weekly data and detailed sociodemographic characteristics of these cases were not available. And the study could not evaluate the change in the cases numbers of symptomatic and asymptomatic STD cases for each route of transmission separately. Second, because China's routine reporting system for notifiable infectious diseases only reported the confirmed cases, and cases could be reported from different medical institutions, the total numbers of tests for different diseases were not available. However, previous studies reported that the lockdown brought a great impact on testing and other services, as well as patients' willingness to visit health services (9–13); we can infer that the minimum number of tests was in February, and then recovered gradually. Third, some other STDs, such as chlamydia, were not included in the analysis because they are not the notifiable infectious diseases in China and are not reported through the surveillance system. In future studies, more provincial-level and city-level surveys are needed to include more kinds of STDs and evaluate the influence of the COVID-19 epidemic on STDs by different severities of the disease and the routes of infection. In further surveys, more sociodemographic information of cases and changes in testing ability during the COVID-19 epidemic of different medical institutions are essential to be investigated.

In conclusion, this is a pioneering study to explore the epidemic of HIV/AIDS and other main STDs under the influence of COVID-19 in China. We found that the case numbers of five STDs showed sharp declines in early 2020, especially in February, compared to the same period in the previous years as well as the predicted case numbers of 2020 based on the ARIMA model. After March 2020, the number of STD cases returned to normal quickly. The epidemic of five STDs was significantly influenced by COVID-19 control measures. These findings indicated that the severe impact of COVID-19 on STDs in China was short-lived, and for providing better STD prevention and control, the effective control of COVID-19 is vital. Our findings could provide references for the development of policies and control measures for STDs under the situation of the COVID-19 epidemic.

## Data Availability Statement

The original contributions presented in the study are included in the article/Supplementary Material, further inquiries can be directed to the corresponding authors.

## Author Contributions

XY, XW, and ZJ were responsible for the study design. XY and XW contributed to the data collection, collation, and writing the report. XY, XZ, LW, and BZ contributed to the data analysis. All authors contributed to the article and approved the submitted version.

## Funding

This work was supported by the National Natural Science Foundation of China (72174004, 91546203, 91846302, and 72104008). The funders had no role in study design, data collection, and analysis, decision to publish, or preparation of the manuscript.

## Conflict of Interest

The authors declare that the research was conducted in the absence of any commercial or financial relationships that could be construed as a potential conflict of interest.

## Publisher's Note

All claims expressed in this article are solely those of the authors and do not necessarily represent those of their affiliated organizations, or those of the publisher, the editors and the reviewers. Any product that may be evaluated in this article, or claim that may be made by its manufacturer, is not guaranteed or endorsed by the publisher.
