# Supplementary material for: The Epidemic of Sexually Transmitted Diseases Under the Influence of COVID-19 in China
Source: Front Public Health. 2021 Dec 16;9:737817. doi: 10.3389/fpubh.2021.737817 (PMC8716580; doi:10.3389/fpubh.2021.737817)
Supplement: Supplementary file 1 [file Data_Sheet_1.DOCX]

**Supplementary files**


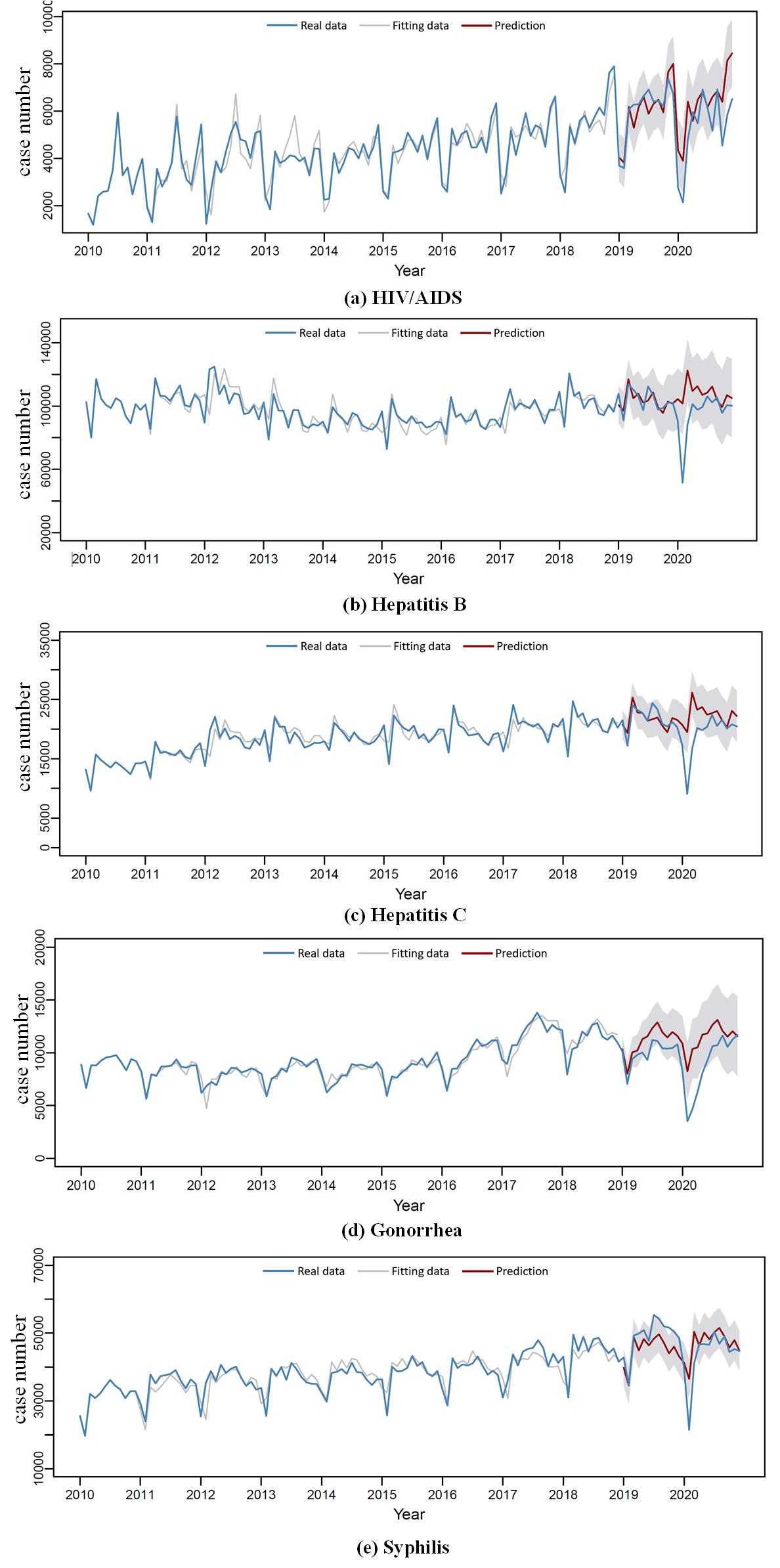


**Supplementary Figure 1. ARIMA model fitting and prediction of the five STDs’ newly reported case number** **in sensitivity analysis**

**Supplementary Table 1. Parameters and goodness-of-fit of the five STDs’ optimal ARIMA models** **in sensitivity analysis**

| **Disease** | **Optimal model** | **Goodness-of-fit** | | | |  | **Ljung-Box test** | |
| --- | --- | --- | --- | --- | --- | --- | --- | --- |
|  |  | **RMSE** | **MAPE (%)** | **AIC** | **BIC** |  | ***χ ^2^* value** | ***P* value** |
| AIDS | ARIMA(1,0,1)×(1,1,0)_12_ * | 480.22 | 9.50 | 1481.39 | 1494.21 |  | 0.128 | 0.72 |
| Hepatitis B | ARIMA(3,1,0)×(2,1,0)_12_ | 5599.83 | 3.70 | 1944.1 | 1959.43 |  | 0.002 | 0.97 |
| Hepatitis C | ARIMA(2,1,1)×(2,1,0)_12_ | 1121.00 | 4.14 | 1640.84 | 1656.16 |  | 0.011 | 0.92 |
| Gonorrhea | ARIMA(2,1,0)×(2,1,0)_12_ | 560.10 | 4.45 | 1499.62 | 1512.39 |  | 0.002 | 0.97 |
| Syphilis | ARIMA(0,0,0)×(2,1,0)_12_ * | 2548.17 | 4.86 | 1801.55 | 1811.81 |  | 0.060 | 0.81 |

Note: RMSE: root mean square error, MAPE: mean absolute percentage error, AIC: Akaike’s information criterion, BIC: Bayesian information criterion.

* Draft was included in the model

**Supplementary Table 2. Each month’s actual case number, predicted number, and absolute percentage error (APE) of five STDs in 2020** **in sensitivity analysis**

| **Month** | **AIDS** | | | **Hepatitis B** | | | **Hepatitis C** | | | **Gonorrhea** | | | **Syphilis** | | |
| --- | --- | --- | --- | --- | --- | --- | --- | --- | --- | --- | --- | --- | --- | --- | --- |
|  | **Actual** | **Predicted** | **APE(%)** | **Actual** | **Predicted** | **APE(%)** | **Actual** | **Predicted** | **APE(%)** | **Actual** | **Predicted** | **APE(%)** | **Actual** | **Predicted** | **APE(%)** |
| January | 2759 | 4333.4 | -57.1 | 91026 | 104452.1 | -14.7 | 17287 | 20706.4 | -19.8 | 8254 | 10861.8 | -31.6 | 39671 | 41222.9 | -3.9 |
| February | 2133 | 3898.6 | -82.8 | 51506 | 101605.8 | -97.3 | 9068 | 19491.1 | -114.9 | 3524 | 8242.9 | -133.9 | 21448 | 36498.4 | -70.2 |
| March | 4808 | 6402.1 | -33.2 | 88150 | 122518.6 | -39.0 | 16718 | 26147.4 | -56.4 | 4661 | 10354.2 | -122.1 | 41154 | 50370.5 | -22.4 |
| April | 5960 | 5585.4 | 6.3 | 101262 | 109474.4 | -8.1 | 20179 | 23251.2 | -15.2 | 6267 | 10502.5 | -67.6 | 46728 | 46365.0 | 0.8 |
| May | 5484 | 6481.5 | -18.2 | 97651 | 112567.5 | -15.3 | 19821 | 23716.1 | -19.7 | 8104 | 11719.9 | -44.6 | 46753 | 50165.1 | -7.3 |
| June | 6915 | 6823.0 | 1.3 | 99319 | 107036.1 | -7.8 | 20367 | 22422.8 | -10.1 | 9292 | 11840.3 | -27.4 | 46538 | 48178.3 | -3.5 |
| July | 6124 | 6166.8 | -0.7 | 106135 | 108443.8 | -2.2 | 22400 | 22690.9 | -1.3 | 10621 | 12655.4 | -19.2 | 50386 | 50248.9 | 0.3 |
| August | 5166 | 6598.5 | -27.7 | 102304 | 112397.0 | -9.9 | 20520 | 23044.7 | -12.3 | 10724 | 13111.3 | -22.3 | 46838 | 51528.3 | -10.0 |
| September | 6927 | 6856.1 | 1.0 | 105377 | 103879.5 | 1.4 | 21538 | 21520.7 | 0.1 | 11643 | 12063.3 | -3.6 | 48965 | 49044.0 | -0.2 |
| October | 4546 | 6392.9 | -40.6 | 95633 | 99321.4 | -3.9 | 20067 | 20337.4 | -1.3 | 10551 | 11520.0 | -9.2 | 44438 | 45702.6 | -2.8 |
| November | 5824 | 8138.6 | -39.7 | 100561 | 106912.2 | -6.3 | 20801 | 23077.2 | -10.9 | 11260 | 12032.2 | -6.9 | 45305 | 47939.3 | -5.8 |
| December | 6508 | 8447.6 | -29.8 | 100209 | 105037.4 | -4.8 | 20438 | 22165.7 | -8.5 | 11691 | 11554.2 | 1.2 | 44696 | 44812.1 | -0.3 |
